# Wiskott-Aldrich syndrome gene as a prognostic biomarker correlated with immune infiltrates in clear cell renal cell carcinoma

**DOI:** 10.3389/fimmu.2023.1102824

**Published:** 2023-04-12

**Authors:** Guixin Ding, Tianqi Wang, Shangjing Liu, Zhongbao Zhou, Jian Ma, Jitao Wu

**Affiliations:** ^1^ Department of Urology, Yantai Yuhuangding Hospital, Qingdao University, Yantai, Shandong, China; ^2^ Department of Urology, Beijing Tiantan Hospital, Capital Medical University, Beijing, China

**Keywords:** clear cell renal cell carcinoma, Wiskott-Aldrich syndrome (WAS) gene, prognosis, drug sensitivity, tumor immune microenvironment

## Abstract

**Introduction:**

The abnormal expression of the Wiskott-Aldrich syndrome protein (WASP) encoded by the Wiskott-Aldrich syndrome (WAS) gene has been implicated in tumor invasion and immune regulation. However, prognostic implications of WAS and its correlation tumor infiltrating in renal clear cell carcinoma (ccRCC) is not clear cut.

**Methods:**

The correlation between WAS expression, clinicopathological variables and clinical outcomes were evaluated using The Cancer Genome Atlas (TCGA), Gene Expression Omnibus (GEO), Tumor Immune Estimation Resource (TIMER), UALCAN, Gene Expression Profiling Interaction Analysis (GEPIA), Kaplan-Meier (KM) plotter and other databases. Furthermore, we assessed the transcription expression of WAS in renal cancer tissues, various renal carcinoma cell lines and human renal tubular cells (HK2) using quantitative polymerase chain reaction (qPCR). A comprehensive analysis of multiple databases including TIMER, GEPIA, TISIDB, ESTIMATE algorithm, and CIBERSORT algorithm were performed to determine the correlation between WAS and tumor infiltrating immune cells in ccRCC.

**Results:**

The results displayed an increase in WAS mRNA level in ccRCC compared to normal tissue. WAS protein level was found highly expressed in cancer tissues, particularly within renal tumor cells via the human protein atlas (HPA). Interestingly, we found that elevated WAS expression was significantly positively correlated with the infiltration of CD8+ T cells, B cells, Monocytes, Neutrophils, Macrophages, T cell regulation, NK cells, and Dendritic cells in ccRCC. Bioinformatics demonstrated a strong correlation between WAS expression and 42 immune checkpoints, including the T cell exhaustion gene PD-1, which is critical for exploring immunotherapy for ccRCC. We revealed that patients with high WAS expression were less sensitive to immunotherapy medications.

**Conclusion:**

In conclusion, our study identified that WAS was a prognostic biomarker and correlated with immune infiltrates in ccRCC.

## Introduction

Renal cell carcinoma (RCC) is one of the most common malignant tumors in the world, accounting for about 2-3% of all malignancies each year (1) and its incidence rate is constantly growing (2). RCC is classified into kidney renal clear cell carcinoma (ccRCC), kidney chromophobe (KICH), and kidney renal papillary cell carcinoma (KIRP). Among them, ccRCC is the most common type, comprising 75-80% of all pathological types (3). ccRCC remains restricted to surgical resection in the early stage, because of pronounced resistance to chemotherapy and radiation therapy (4, 5). ccRCC patients seldom be diagnosed in early stage due to the lack of reliable biomarkers. It is estimated that up to 20%-30% of ccRCC patients suffer from recurrence or metastasis after radical nephrectomy (6). Although immune checkpoint inhibitors (ICIs) have been applied to treat metastatic ccRCC patients and shown improved prognosis, the efficacy varies from individual (7). Consequently, the novel immune related biomarkers that improve targeting for early diagnosis of ccRCC are particularly urgent.

It is reported that renal cancer often exhibits with inflamed but immunosuppressive tumor microenvironment (TME). The development and progression of tumor depend on other cells in TME, particularly immune cells (8). Studies have demonstrated that the immune infiltration of macrophages and T cells in tumors may have a direct impact on the outcome of ccRCC patients (9–13). Thus, explicating the immune infiltrate pattern of ccRCC holds promise for improving immunotherapy efficacy.

The WAS (Wiskott-Aldrich syndrome) gene was the product of a mutant gene originally found in Wiskott-Aldrich syndrome, a recessive X-linked immunodeficiency disease (14). The Wiskott-Aldrich syndrome protein (WASP), encoded by the WAS gene, is one of actin regulatory proteins. A variety of cellular functions are carried out by WASP, including actin cytoskeleton-based regulation, cell movement, signal transduction, and others (15–17). Furthermore, WASP may also mediate tumor invasion and metastasis *via* inhibiting cell-cell adhesion, destroying extracellular matrix, and promoting the formation of pseudopods (18–20). Several studies have linked the abnormal expression of WAS to tumor invasion and immune regulation in multiple cancer, including chronic myeloid leukemia, lymphoma, breast cancer, and prostate cancer (21–23). Whereas, the oncogenicity and clinical significance of WAS in ccRCC remained uncertain.

In this study, we assessed the aberrant expression of WAS in human malignancies, the prognosis of ccRCC and the link between clinical pathology through databases such as TCGA, GEO, Gene Expression Profiling Interactive Analysis (GEPIA), UALCAN datasets and Kaplan-Meier (KM) plotter. Then, the correlation between WAS expression and tumor-infiltrating immune cells/marker genes in ccRCC was investigated using multiple databases, including TIMER, GEPIA, and TISIDB. Additionally, we explored WAS-interacting protein networks on the STRING website and performed enrichment analyzes for co-expressed genes. These findings might shed light on the immunomodulatory effects of WAS on ccRCC.

## Materials and methods

### The cancer genome atlas database

The TCGA database (https://genome-cancer.ucsc.edu/) is an open and freely accessible online database of cancer genomes consisting of clinical and pathological data on 30+ different cancer types. Through the TCGA database, we were able to gather information on ccRCC patients, including RNA-seq expression, prognosis, and pertinent clinicopathological information. The correlation of WAS and immune infiltration in ccRCC was observed by ESTIMATE and ssGSEA analysis. Median RNA expression was used as a cutoff to stratify patients into elevated or low expression groups, while the area under the receptor operating characteristic (ROC) curve (AUC) was applied for qualitative and quantitative performance measure.

### The gene expression omnibus database

The National Biotechnology Information Center (NBIC) (https://www.ncbi.nlm.nih.gov/geo/) maintains the GEO database, which is an extensive gene expression database. Transcriptomic data was obtained from the GEO (GSE53757), an independent validation cohort of ccRCC patients.

### UALCAN, and clinical proteomic tumor analysis consortium

UALCAN is a convenient and easy-to-use online application that can be used for analyzing publicly available information about cancer (24). Proteomics technologies enable CPTAC to characterize and quantify tumor samples by mass spectrometry, which is able to identify each tumor sample’s characteristic proteome and constituent proteins. UALCAN was used in this study to analyze the flux of WAS protein expression obtained from CPTAC.

### The human protein atlas database

The HPA database (https://www.proteinatlas.org/) provides information on 26,000 human proteins, as well as their distribution in tissues and cells (25). With the use of specific antibodies, it is possible to visualize the expression profiles of proteins in normal tissues, cancer cells and cell lines by immunohistochemistry. In this study, we investigated the expression of WAS in both normal and tumor tissues by utilizing the HPA database.

### Kaplan-Meier plotter

The KM plotter can draw survival curves using 530 ccRCC samples to assess survival prognosis of related genes (26). In ccRCC, this database was used to asses overall survival (OS) and progression-free survival (PFS) of WAS in addition to estimating hazard ratios (HR) and p-values.

### Protein-protein interaction network and functional enrichment analysis

STRING (https://cn.string-db.org/) is an online tool for searching and constructing PPI interaction networks using interacting genes and proteins. The following main parameters are used to create a WAS co-expression network: 1) Active interaction sources: Co-expression, Co-occurrence, Textmining, Experiments, Databases; 2) The meaning of the network edge: evidence; 3) Maximum number of interactions: 10; 4) Minimum Required Interaction Score: High Confidence (0.700). Gene Ontology (GO) enrichment and Kyoto Encyclopedia of KEGG pathway analysis of coexpression genes were performed with the ClusterProfiler package, the results of which were visualized using “ggplot2”.

### Tumor immune estimation resource database

Tumor immune estimation resource (TIMER2.0) (https://timer.cistrome.org/) is an online data platform, which can be used to systematically analyze the immune infiltration in various malignant tumors (27). In addition, tumor purity can be estimated from this database. “GENE” module was applied to examine the WAS expression and its associations with tumor-infiltrating lymphocyte (TIL). Expression scatter plots were created by the correlation module for the correlation and estimated statistical significance of WAS expression with immune cell marker genes.

### The gene expression profiling interactive analysis analysis

The Gene Expression Profiling Interactive Analysis (GEPIA) online database (http://gepia.cancer-pku.cn/index.html) is a comprehensive platform consisting of 8587 normal and 9736 tumor samples from GTEx and TCGA data (28). As part of this study, we analyzed the relationship between WAS expression and various immune cell markers in ccRCC as well as OS and progression-free survival (PFS) in ccRCC.

### Tumor-immune system interaction database

In order to study the interaction between tumors and immune factor, TISIDB (http://cis.hku.hk/TISIDB/index.php) combines data sources from a variety of heterogeneous sources. This database can be used for predicting immunotherapy responses and identifying new immunotherapy targets, among others. TISIDB was employed in this study to examine the relationships between WAS and 28 TILs, 45 immunostimulators, 24 immunoinhibitors, 41 chemokines, and 18 receptors in ccRCC (29).

### Differentially expressed genes

Patients with ccRCC in TCGA were divided into high expression and low expression group based on the median expression of WAS. DEGs between two groups were identified by R software “limma” with P < 0.05 and |Log2 (Fold Change)| > 1 as thresholds.

### Immune cell proportion analyses and immune related features

To explore the immune cell abundance in ccRCC tissues, CIBERSORT (30) was employed to evaluate the proportions of 22 immune cell types using a deconvolution algorithm by the R package with default parameters. In addition, the ESTIMATE scores (ES), tumor purity (TP), stromal scores (SS), and immune scores (IS) for each ccRCC sample were evaluated using the ESTIMATE algorithm (31) of the “estimate” package.

### Immunotherapy and chemotherapeutic response in risk score subtype

The immunophenoscore (IPS) is a machine learning-based scoring system applied for the prediction of patients’ responses to ICI treatment based on the weight average Z scores representing immune-related genes expression in cell types (32). High IPS scores reflect increased immunogenicity. As targeted therapy is widely used to treat ccRCC, risk scores were used to predict the drug sensitivity based on half-maximal inhibitory concentrations (IC50) for each ccRCC patient from the Genomics of Drug Sensitivity in Cancer (GDSC) website (33) using the R package “pRRophetic” (34).

### Cell lines and cell culture

Renal tubular epithelial cell line (HK-2) and ccRCC cell line (ACHN, Caki-2, 786-O, 769-P, A498) were purchased from Cell bank of Chinese Academy of Sciences. The HK-2 cells were cultured with DMEM (BI, Israel) and the other cells with RPMI1640 (BI, Israel). There was 10% fetal bovine serum (FBS) added to all media, as well as 1% penicillin and streptomycin. A humidified incubator with 5% carbon dioxide and 37°C was used for culture of each cell type.

### Sample collection

Approved by the ethics committee of the affiliated Yantai Yuhuangding hospital of Qingdao university, a total of 20 eligible ccRCC patients were enrolled. ccRCC samples were acquired from patients who underwent radical nephrectomy, or partial nephrectom. The pathological specimens were confirmed by two independent pathologists.

### RNA extraction and quantitative real-time PCR

Total RNA was extracted with Trizol reagent (Pufei, Shanghai) according to the manufacturer’s instructions. RNA was reverse transcribed to cDNA using the Promega M-MLV kit. The primer sequence of WAS for qPCR is forward 5′-CACGAGAACCAGCGACTCTTTG-3′ and reverse 5′-CCACAATGCTCCTTGGTCCAGT-3′.

### Statistical analysis

We carried out all statistical analyzes with R (version 4.2.1) and visualizing the results with ggplot2 (version 3.3.3). In order to determine whether there are any differences between ccRCC tissues and normal surrounding tissues, the Mann-Whitney U test and paired t-test were performed. Execute the KM graph to build the survival curve. For KM plot, GEPIA, and TISIDB, HR, and *P* values are described using the log-rank test. In order to determine the relationship of WAS expression with immune infiltration levels, immunomodulators and chemokines, spearman’s correlation coefficients were calculated. A very weak correlation was determined by <0.2, a weak correlation by < 0.4, a moderate correlation by < 0.6, a strong correlation by < 0.8, and a very strong correlation by < 1.0. Statistical significance was determined by *P* < 0.05.

## Result

### Aberrant WAS expression in ccRCC

**Figure 1:**
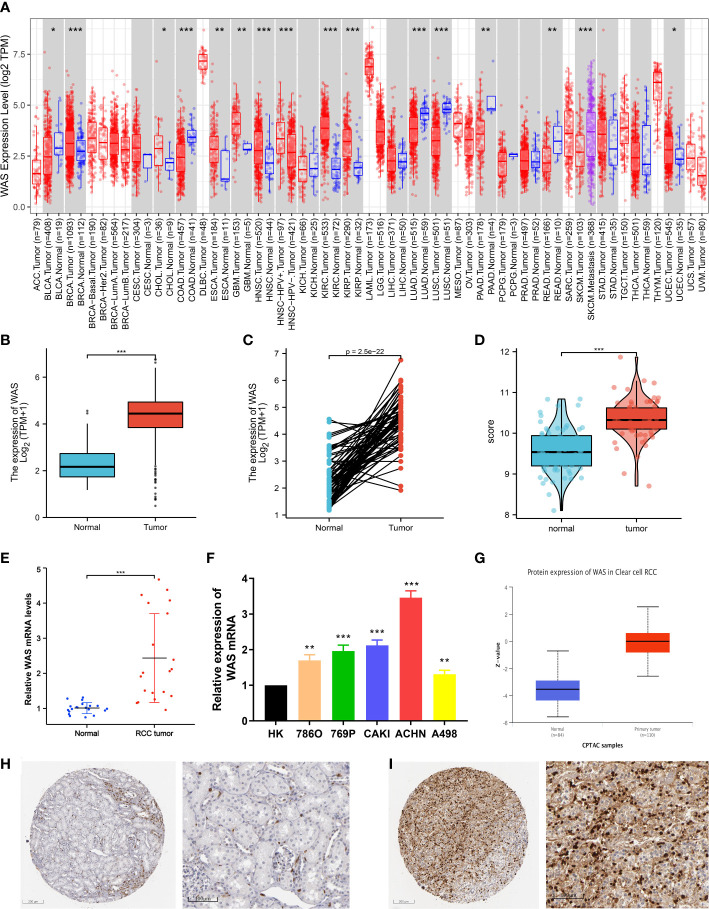
**(A)** Increased or decreased WAS in different tumor types from The Cancer Genome Atlas (TCGA) database were determined by Tumor Immune Estimation Resource (TIMER) (*P < 0.05, **P < 0.01, ***P < 0.001). **(B)** The mRNA expression level of was in 539 ccRCC samples in TCGA database was higher than that in 72 normal samples (*P* < 0.001). **(C)** Increased WAS expression in ccRCC compared with the matching normal tissue from TCGA database (n = 72). **(D)** The mRNA expression level of WAS in tumor samples matched in GEO database was higher than that in adjacent normal samples (*P* < 0.001). **(E)** PCR experiment on matched samples of 20 eligible ccRCC patients. **(F)** PCR experiment to verify the expression of WAS gene in renal cell carcinoma cell lines. **(G)** In the UALCAD database, the expression of WAS protein in ccRCC was higher than that in normal tissues. **(H)** WAS expression levels in normal kidney tissues detected by IHC through HPA database. **(I)** WAS expression levels in renal carcinoma tissues detected by IHC through HPA database.

The pan-cancer analysis demonstrated that WAS expression was elevated in most cancer types, including breast invasive carcinoma, head and neck squamous cell carcinoma, renal clear cell carcinoma, and renal papillary cell carcinoma compared with adjacent tissues (**Figure 1A**).

To determine the mRNA and protein expression of WAS in ccRCC, the expression profile from TCGA, GEO, and UALCAD was analyzed. Our analysis of **Figure 1B** shows that the WAS mRNA in ccRCC samples (539 cases) of TCGA was higher than that in the normal samples (72 cases) (*P <* 0.001), which was similar to the data from TIMER database. From the TCGA database, **Figure 1C** represents that WAS expression in ccRCC was higher than that in matched normal tissues (n = 72). The same information can also be verified in the GEO database (GSE53757) (**Figure 1D**). By using the CPTAC analysis of UALCAN, we were able to conduct the protein expression analysis of WAS. The results showed that protein expression of WAS (n = 110) in ccRCC was significantly higher than that in normal tissues (n = 84) (**Figure 1G**). Furthermore, qPCR was performed to examine the expression of WAS in 20 pairs of ccRCC cases and adjacent normal tissues. At the same time, we verified that WAS gene expression in most ccRCC cell lines was higher than that in the control group through qPCR experiment (**Figure 1F**). It was observed that WAS mRNA (**Figures 1E, F**) and protein levels (**Figure 1G**) were higher in most ccRCC tissues than in paired adjacent normal tissues. Validation of protein expression was conducted using Human Protein Atlas (HPA) database (**Figures 1H, I**). And we found elevated expression levels of WAS protein in cancer tissues, particularly within renal tumor cells. Based on the above data, WAS gene was increased significantly in ccRCC, which might indicate that it can be used as a potential diagnostic biomarker.

### Associations between WAS levels and clinical pathological features

**Figure 2:**
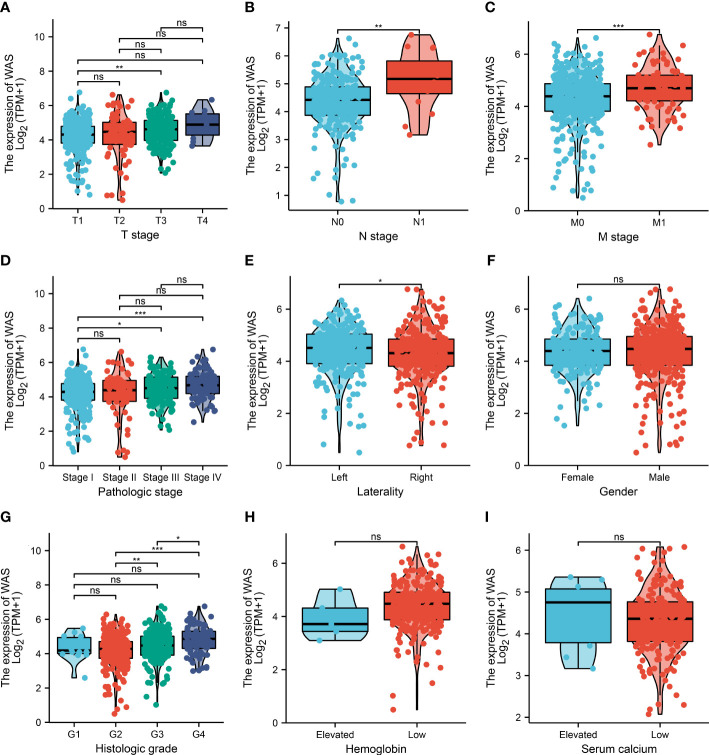
The relationship between WAS expression and clinicopathological features. Correlation analysses between WAS mRNA expression and T stage **(A)**, lymph node metastasis **(B)**, M stage **(C)**, pathological stage **(D)**, laterality **(E)**, gender **(F)**, histological grade **(G)** hemoglobin **(H)** and serum calcium **(I)**. (ns: no significance; *P < 0.05, **P < 0.01, ***P < 0.001).

**Table 1** describes the baseline characteristics of ccRCC patients. As is shown in **Table 1** and **Figure 2**, elevated expression of WAS was significantly correlated with distant metastasis, tumor stage, pathological stage, histological grade, laterality, and overall survival (OS) (*P* < 0.05). The association between WAS expression and other clinicopathological features, such as gender, serum calcium level, hemoglobin, and age, was not statistically significant (*P* > 0.05). In general, these results indicated that WAS expression level was associated with TNM stages, pathological/histological grade, and prognosis of ccRCC patients.

**Table 1:** The characteristics of ccRCC patients included in this study.

Characteristic	Low expression of WAS	High expression of WAS	p
n	269	270	
T stage, n (%)			0.045
T1	153 (28.4%)	125 (23.2%)	
T2	35 (6.5%)	36 (6.7%)	
T3	78 (14.5%)	101 (18.7%)	
T4	3 (0.6%)	8 (1.5%)	
N stage, n (%)			0.090
N0	121 (47.1%)	120 (46.7%)	
N1	4 (1.6%)	12 (4.7%)	
M stage, n (%)			0.008
M0	227 (44.9%)	201 (39.7%)	
M1	28 (5.5%)	50 (9.9%)	
Pathologic stage, n (%)			0.014
Stage I	152 (28.4%)	120 (22.4%)	
Stage II	30 (5.6%)	29 (5.4%)	
Stage III	56 (10.4%)	67 (12.5%)	
Stage IV	30 (5.6%)	52 (9.7%)	
Gender, n (%)			0.397
Female	98 (18.2%)	88 (16.3%)	
Male	171 (31.7%)	182 (33.8%)	
Age, n (%)			0.093
<=60	124 (23%)	145 (26.9%)	
>60	145 (26.9%)	125 (23.2%)	
Histologic grade, n (%)			< 0.001
G1	8 (1.5%)	6 (1.1%)	
G2	133 (25%)	102 (19.2%)	
G3	99 (18.6%)	108 (20.3%)	
G4	22 (4.1%)	53 (10%)	
Serum calcium, n (%)			0.726
Elevated	4 (1.1%)	6 (1.6%)	
Low	108 (29.5%)	95 (26%)	
Normal	79 (21.6%)	74 (20.2%)	
Hemoglobin, n (%)			0.141
Elevated	4 (0.9%)	1 (0.2%)	
Low	123 (26.8%)	140 (30.5%)	
Normal	103 (22.4%)	88 (19.2%)	
OS event, n (%)			0.011
Alive	197 (36.5%)	169 (31.4%)	
Dead	72 (13.4%)	101 (18.7%)	
Age, median (IQR)	61 (53, 71)	59.5 (51, 69)	0.093

### The expression of WAS was a potential biomarker for ccRCC

**Figure 3:**
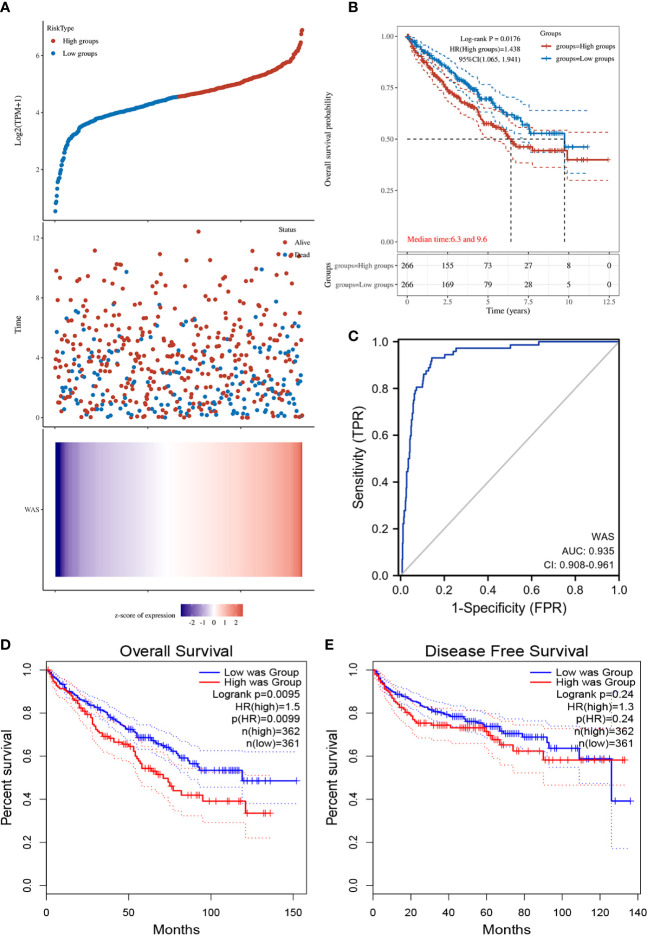
**(A)** WAS expression distribution and survival status. 0: dead, 1: alive. The blue dots represent the surviving ccRCC patients, and the red dots represent the dead ccRCC patients. **(B)** Kaplan-Meier survival curves indicated that ccRCC patients with high WAS mRNA expression had a shorter OS. **(C)** Diagnostic ROC curve of WAS. **(D)** The relationship between WAS expression and OS was obtained by GEPIA. **(E)** The relationship between WAS expression and DFS of ccRCC was obtained by GEPIA. OS, overall survival; DFS, disease free survival.

The distribution of WAS expression, survival status of ccRCC patients, and expression profiles of WAS were shown in **Figure 3A**. With the increase of risk score in ccRCC patients, the number of dead ccRCC patients increased gradually. Additionally, the Kaplan-Meier survival curves indicated that high-WAS patients had poorer survival and a higher risk of death than those of low-WAS patients (*P* = 0.0176) (**Figure 3B**). The ROC analysis demonstrated that the elevated WAS expression allowed for highly accurate ccRCC diagnosis (AUC = 0.935, 95% CI: 0.908–0.961) (**Figure 3C**). Additionally, we verified that WAS expression was correlated with OS in ccRCC using GEPIA (*P* < 0.01) (**Figure 3D**). A significant correlation between WAS expression and DFS (Disease Free Survival) was not detected (**Figure 3E**).

### DEGs analysis

**Figure 4:**
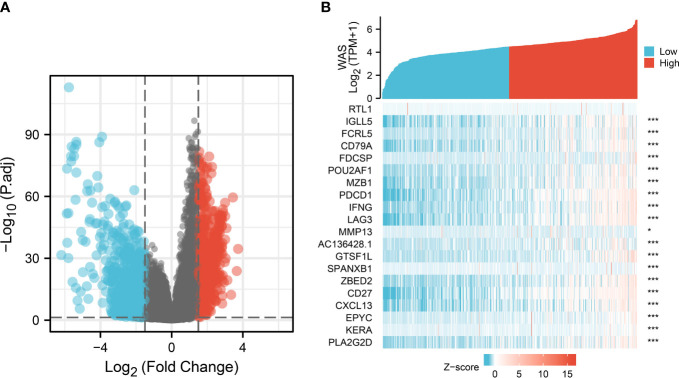
DEGs between patients with high and low WAS expression. **(A)** Volcano plot of differentially expressed genes between high and low WAS expression groups. **(B)** Heatmap of the top twenty significantly differentially expressed protein-coding genes in the two groups. Red and blue dots represent up- and down-regulated genes, respectively. DEG, differentially expressed genes.

DEG analysis between the high- and low-WAS groups in the TCGA cohort showed 475 up-regulated and 1020 down-regulated DEGs (**Figure 4A**). The heatmap suggested the top twenty significant up- and down-regulated genes (**Figure 4B**).

### Enrichment analysis of WAS in ccRCC

**Figure 5:**
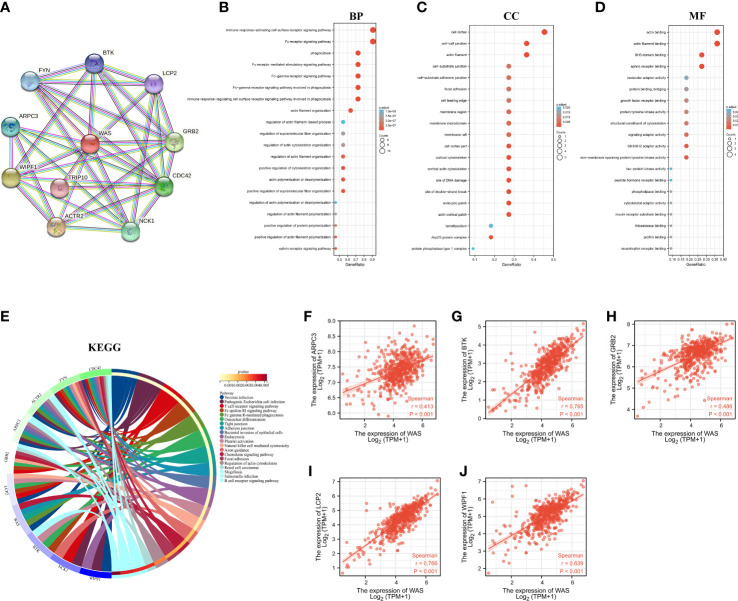
PPI network and functional enrichment analysis. **(A)** WAS and its co-expressed gene network. BP **(B)**, CC **(C)**, MF **(D)**, KEGG **(E)** enrichment analysis of 10 related genes. **(F–J)** Correlation analysis of WAS expression with co-expressed genes in ccRCC. BP, Biological Process; CC, Celluar Components; MF, Molecular Functtion; KEGG, Kyoto Encylopedia of Genes and Genomes; ccRCC, clear cell renal cell carcinoma.

An interactive map was created between WAS and its 10 co-expressed genes based on the STRING database, and constructed a PPI network based on this map (**Figure 5A**). The functional annotations in **Figure 5B** show that WAS is related to Fc receptor signaling pathway, immune response-activating cell surface receptor signaling pathway, immune response-regulating cell surface receptor signaling pathway involved in phagocytosis, Fc-gamma receptor signaling pathway involved in phagocytosis, Fc-gamma receptor signaling pathway, phagocytosis, and so on. **Figures 5C, D** illustrate the components of cells as well as their molecular functions. KEGG pathway analysis results showed that the top 10 related genes shown in the PPI network are involved in T cell receptor signaling pathway, Fc gamma R-mediated phagocytosis, tight junction, adherens junction, endocytosis, natural killer cell mediated cytotoxicity, renal cell carcinoma and B cell receptor signaling pathway, etc (**Figure 5E**). As is presented in **Figures 5F–J**, the WAS expression level was positively correlated with ARPC3 (Actin Related Protein 2/3 Complex Subunit 3) (*r* = 0.413, *P* < 0.001), BTK (Bruton Tyrosine Kinase) (*r* = 0.765, *P* < 0.001), GRB2 (Growth Factor Receptor Bound Protein 2) (*r* = 0.486, *P* < 0.001), LCP2 (Lymphocyte cytosolic protein 2) (*r* = 0.766, *P* < 0.001), and WIPF1 (WAS/WASL Interacting Protein Family Member 1) (*r* = 0.639, *P* < 0.001).

### Correlation between immune infiltration and WAS expression

**Figure 6:**
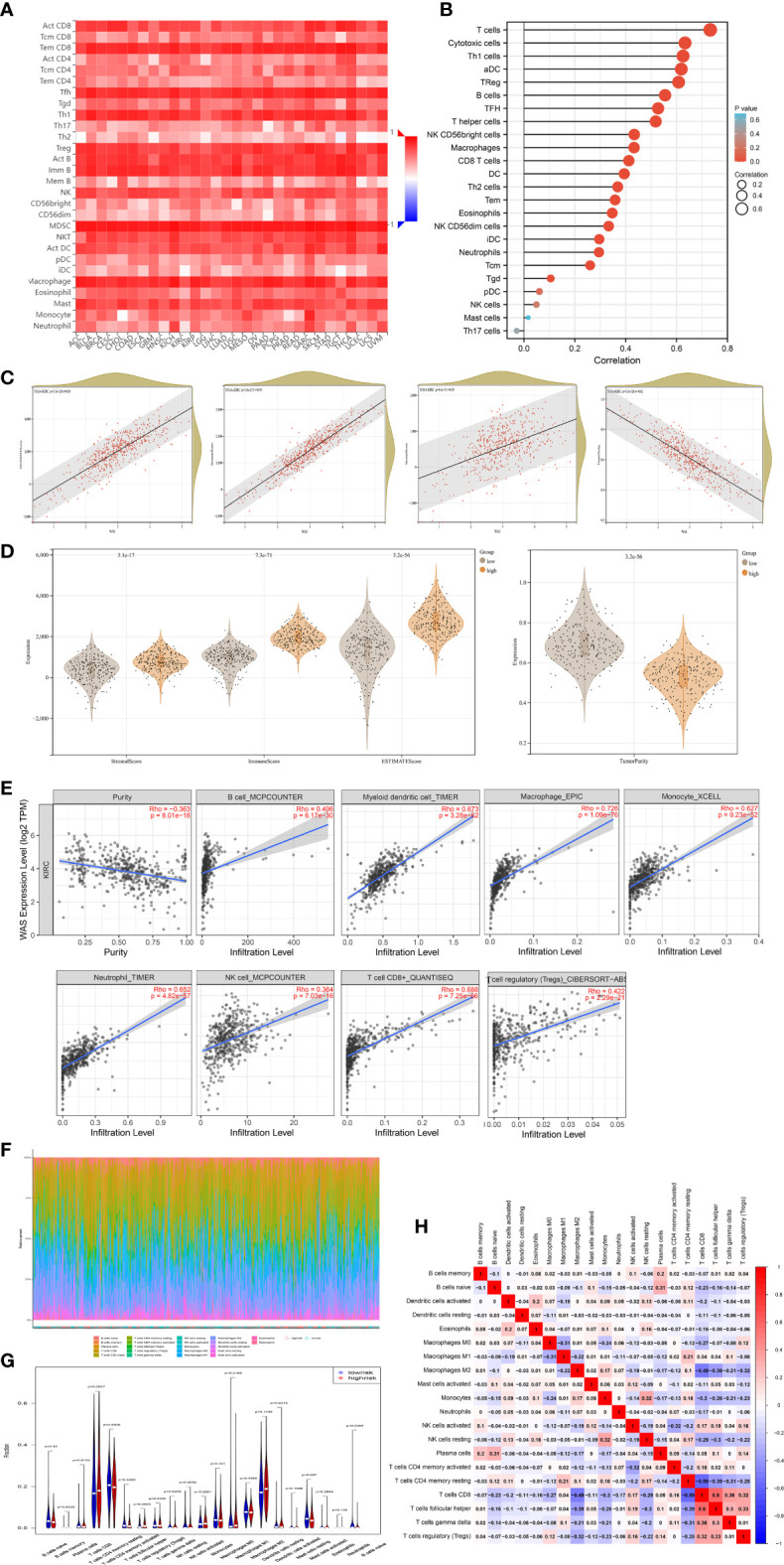
Correlation of WAS expression and immune infiltration in ccRCC. **(A)** Correlation between the expression of WAS and the abundance of TILs in ccRCC in the TISIDB database. **(B)** ssGSEA analysis identified multiple types of immune cell infiltration levels in TCGA ccRCC samples to correlate with WAS. **(C)** ESTIMATE algorithm showed that WAS expression in ccRCC was positively correlated with stroma and immune scores. **(D)** Stromal score, immune score, ESTIMATE score and tumor purity was calculated by ESTIMATE algorithm. **(E)** WAS expression in ccRCC correlated with infiltration levels of CD8+ T cells, Treg cells, B cells, neutrophils, macrophages, bone marrow dendritic cells, natural killer cells and monocytes, available in the TIMER2.0 database. **(F, G)** The specific 22 immune fractions represented by various colors in each sample through CIBERSORT algorithm were shown in barplot and violin. **(H)** Correlation between 21 types of immune cells. TILs, tumor-infiltrating lymphocytes; TIMER2.0, tumor immune estimation resource. Color images are available online.

To determine whether WAS expression was correlated with immune cell infiltration in ccRCC, we employed the TIMER2.0 online platform and the ssGSEA algorithm. Genetic techniques used to study immune infiltration must take into account the purity of tumor cells in clinical cancer samples. In our research, WAS expression was adversely correlated with the purity of ccRCC (*r* = -0.363, *P* < 0.001). Additionally, we observed a strong correlation between WAS and TIL abundance (**Figure 6A**). We used the ssGSEA algorithm to identify the levels of immune cell infiltration in TCGA ccRCC samples, and the vast majority of which were positively correlated with WAS expression (**Figure 6B**). The ESTIMATE algorithm revealed a profound association of WAS with stromal score (*r* = 0.50, *P* < 0.001), immune score (*r* = 0.93, *P* < 0.001), ESTIMATE score (*r* = 0.83, *P* < 0.001), and tumor purity (*r* = -0.82, *P* < 0.001) in ccRCC (**Figure 6C**). As predicted by the ESTIMATE algorithm, high WAS expression patients were significantly higher in immune score (*P* < 0.001), stromal score (*P* < 0.001), and ESTIMATE score (*P* < 0.001) compared with low WAS expression patients (**Figure 6D**). In contrast, the ESTIMATE algorithm was able to determine that elevated WAS expression groups had a lower tumor purity than low WAS expression group (**Figure 6D**). For example, the correlation between WAS and T cells, cytotoxic cells, Th1 cells, ADC, Tregs, B cells, TFH, T helper cells, and other immune cells was greater than 0.5. Based on the TIMER database, we found that the WAS expression was correlated with the infiltration of CD8 T cells (*r* = 0.688), B cells (*r* = 0.496), monocytes (*r* = 0.627), neutrophils (*r* = 0.652), macrophages (*r* = 0.726), T cell regulation (*r* = 0.422), NK cells (*r* = 0.364) and bone marrow dendritic cells (*r* = 0.673) (**Figure 6E**). All p-values are much less than 0.001. Additionally, Box and violin plot showed the specific fraction of 22 immune cells in each ccRCC sample by the CIBERSORT algorithm (**Figures 6F–H**). These results suggested that WAS was critically correlated with ccRCC immune infiltration.

To further confirm the relationship between WAS expression and immune cell infiltration levels in ccRCC, the association between WAS and different biomarkers of TILs (CD8+/CD4+ T cells, NK cells, B cells, monocytes, DCs, TAMs, M1 macrophages, M2 macrophages, neutrophils and T cell exhaustion-related subtypes) in ccRCC was investigated using TIMER, TCGA and GEPIA databases. It was found that most TIL markers in ccRCC were associated with WAS. There were also several functional T cells under analysis, including Tfh/Th1/Th2/Th17 cells, Tregs, and exhausted T cells. Particularly, WAS showed a strong association with a majority of immune markers sets of TILs in ccRCC (**Table 2**).

**Table 2:** Correlation analysis between WAS and related genes and markers of immune cells in Tumor Immune Estimation Resource (TIMER2.0).

Description	Gene markers	ccRCC None Cor	p	Purity Cor	p
CD8 T cell+	CD8A	0.716	***	0.682	***
	CD8B	0.706	***	0.68	***
	TBX21	0.549	***	0.521	***
	IFNG	0.655	***	0.61	***
	CXCL9	0.672	***	0.625	***
	CXCL10	0.592	***	0.54	***
T cell (general)	CD3D	0.793	***	0.769	***
	CD3E	0.819	***	0.797	***
	CD3G	0.723	***	0.696	***
	CD2	0.799	***	0.775	***
B cell	CD19	0.534	***	0.493	***
	CD79A	0.578	***	0.537	***
	BLK	0.539	***	0.508	***
Monocyte	CD86	0.786	***	0.763	***
	CD115 (CSF1R)	0.767	***	0.749	***
TAM	CCL2	0.18	***	0.112	*
	CD68	0.493	***	0.497	***
	IL10	0.537	***	0.465	***
	CSF2	0.283	***	0.29	***
M1 Macrophage	INOS (NOS2)	0.078	0.0736	0.011	0.806
	IRF5	0.476	***	0.468	***
	COX2(PTGS2)	0.043	0.319	-0.021	0.651
M2 Macrophage	CD163	0.504	***	0.474	***
	VSIG4	0.599	***	0.561	***
	MS4A4A	0.572	***	0.527	***
Neutrophils	CD66b(CEACAM8)	0.045	0.3	0.05	0.285
	CD11b (ITGAM)	0.674	***	0.644	***
	CCR7	0.62	***	0.584	***
Natural killer cell	KIR2DL1	0.156	***	0.118	*
	KIR2DL3	0.2	***	0.203	***
	KIR2DL4	0.409	***	0.392	***
	KIR3DL1	0.181	***	0.198	***
	KIR3DL2	0.287	***	0.281	***
	KIR3DL3	0.153	***	0.129	**
	KIR2DS4	0.176	***	0.16	***
Dendritic cell	HLA-DPB1	0.809	***	0.809	***
	HLA-DQB1	0.527	***	0.49	***
	HLA-DRA	0.747	***	0.74	***
	HLA-DPA1	0.735	***	0.709	***
	BDCA-1(CD1C)	0.357	***	0.293	***
	BDCA-4(NRP1)	0.033	0.452	-0.042	0.364
	CD11c (ITGAX)	0.649	***	0.624	***
Th1	T -bet (TBX21)	0.549	***	0.521	***
	STAT4	0.59	***	0.531	***
	STAT1	0.592	***	0.546	***
	IFN-γ (IFNG)	0.655	***	0.61	***
	TNF-α (TNF)	0.442	***	0.413	***
Th2	GATA3	0.319	***	0.318	***
	STAT6	0.117	**	0.139	**
	STAT5A	0.666	***	0.633	***
	IL13	0.117	**	0.09	0.0538
Tfh	BCL6	0.083	0.0565	0.081	0.0832
	IL21	0.23	***	0.208	***
Th17	STAT3	0.192	***	0.146	**
	IL17A	0.065	0.133	0.032	0.5
Treg	FOXP3	0.69	***	0.657	***
	CCR8	0.605	***	0.561	***
	STAT5B	0.056	0.199	0.052	0.262
	TGFβ (TGFB1)	0.221	***	0.173	***
T cell exhaustion	PD-1 (PDCD1)	0.771	***	0.755	***
	PDL-1(CD274)	0.227	***	0.198	***
	CTLA4	0.712	***	0.682	***
	LAG3	0.731	***	0.703	***
	TIM-3 (HAVCR2)	0.299	***	0.245	***
	GZMB	0.516	***	0.468	***

ccRCC, renal clear cell carcinoma; TAM, tumor-associated macrophage; Th, T helper cell; Tfh, Follicular helper T cell; Treg, regulatory T cell; Cor, R value of Spearman’s correlation; None, correlation without adjustment; Purity; correlation adjusted by purity.

*p < 0.05; **p < 0.01; ***p < 0.001.

**Figure 7:**
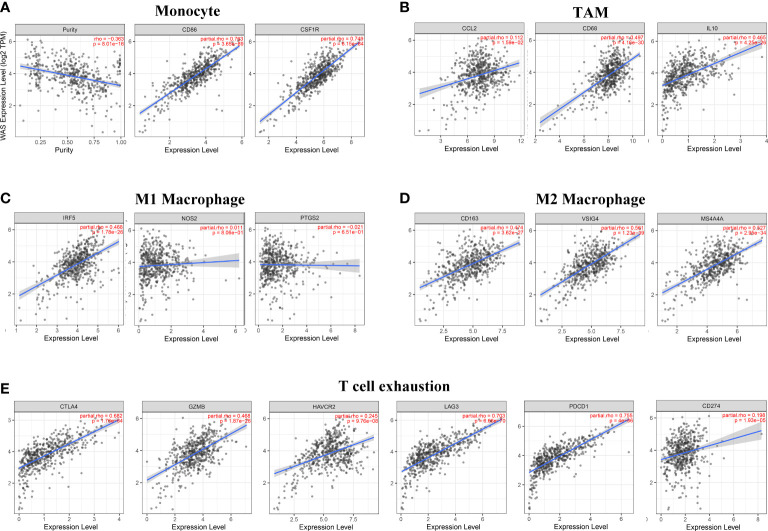
WAS expression is associated with macrophage, monocyte, and T cell exhaustion in clear cell renal cell carcinoma (ccRCC). Markers include CSF1R and CD86 of monocytes; CD68, IL10 and CCL2 of TAM; PTGS2, IRF5 and NOS2 of M1 macrophages; CD163, VSIG4 and MS4A4A of M2 macrophages; and PD-1, PD-L1, CTLA4, LAG3, TIM-3 and GZMB of T cell exhaustion. The following scatterplots illustrate the correlations between WAS expression and gene markers of monocytes **(A)** and TAMs **(B)** as well as M1 **(C)** and M2 macrophages **(D)** and T-cell exhaustion **(E)** in ccRCC.

Obviously, WAS was significantly associated with most marker sets, including monocytes, TAMs, M2 macrophages, and T cell exhaustion (**Table 2**). Our study demonstrated that WAS are markedly correlated with the chemokine ligands of TAMs (IL10, CCL2 and CD68) and T-cell exhaustion (CTLA4, PD-1, PD-L1, GZMB, LAG3, and HAVCR2) in ccRCC, with the same M1 macrophages (IRF5) and M2 macrophages (MS4A4A, CD163, VSIG4) (*P* < 0.001; **Figures 7A–E**). Additionally, based on the GEPIA database, we further assessed the association between WAS expression and the aforementioned markers of M1 macrophages, M2 macrophages, TAMs, monocytes, and T-cell exhaustion. Moreover, the results were similar to those found in TIMER (**Table 3**). Thus, our research suggested that WAS may regulate the exhaustion of T cells and macrophage polarization in ccRCC.

**Table 3:** Correlation analysis between WAS and related genes and markers of monocyte, macrophages, and T-cell exhaustion in Gene Expression Profiling Interaction Analysis (GEPIA).

Description	Gene markers	KIRC Tumor Cor	p	Normal Cor	p
CD8 T cell+	CD8A	0.71	***	0.8	***
	CD8B	0.69	***	0.79	***
	TBX21	0.55	***	0.76	***
	IFNG	0.66	***	0.32	**
	CXCL9	0.68	***	0.58	***
	CXCL10	0.62	***	0.62	***
T cell (general)	CD3D	0.76	***	0.85	***
	CD3E	0.81	***	0.88	***
	CD3G	0.76	***	0.84	***
	CD2	0.79	***	0.86	***
B cell	CD19	0.56	***	0.69	***
	CD79A	0.56	***	0.8	***
	BLK	0.54	***	0.7	***
Monocyte	CD86	0.78	***	0.89	***
	CD115 (CSF1R)	0.79	***	0.86	***
TAM	CCL2	0.18	***	0.28	*
	CD68	0.48	***	0.83	***
	IL10	0.54	***	0.41	***
	CSF2	0.29	***	0.5	***
M1 Macrophage	INOS (NOS2)	0.12	**	0.16	0.17
	IRF5	0.49	***	-0.22	0.061
	COX2(PTGS2)	0.1	*	-0.0062	0.96
M2 Macrophage	CD163	0.58	***	0.81	***
	VSIG4	0.63	***	0.82	***
	MS4A4A	0.61	***	0.86	***
T cell exhaustion	PD-1 (PDCD1)	0.76	***	0.65	***
	PDL-1(CD274)	0.16	***	0.18	0.12
	CTLA4	0.72	***	0.71	***
	LAG3	0.7	***	0.097	0.42
	TIM-3 (HAVCR2)	0.34	***	0.65	***
	GZMB	0.47	***	0.85	***

ccRCC, renal clear cell carcinoma; TAM, tumor-associated macrophages; Normal, correlation analysis in normal tissue of TCGA; Tumor, correlation analysis in tumor tissue of TCGA.

*p < 0.05; **p < 0.01; ***p < 0.001.

Additionally, correlation analysis represented that WAS expression was positively correlated with T-cell exhaustion-related markers (CTLA4, PD-1, PD-L1, LAG3, TIM-3 and GZMB) in ccRCC (*r* = 0.198~0.771, *P <* 0.001) (**Figure 7E**).

### WAS expression was associated with immunomodulators in ccRCC

**Figure 8:**
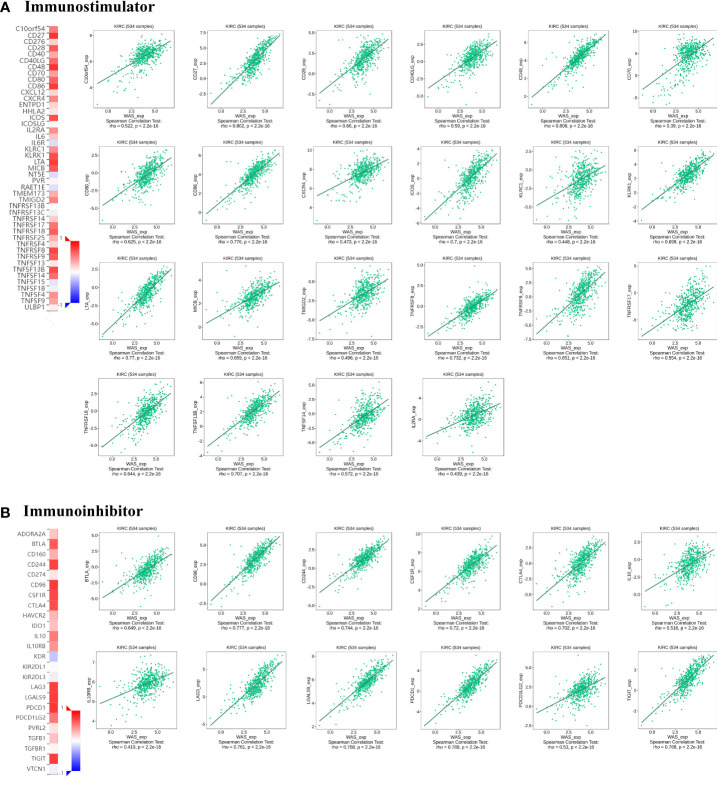
Correlation of WAS expression with immunomodulators in ccRCC. **(A)** Correlation between WAS expression and immunostimulants in ccRCC in the TISIDB database. **(B)** Correlations between WAS expression and immunoinhibitors in ccRCC a in the TISIDB database.

Correlation analyses indicated that WAS expression in ccRCC was significantly correlated with immunostimulants (*P <* 2.2e-16), such as C10orf54 (VSIR) (*r* = 0.522), CD27 (*r* = 0.802), CD28 (*r* = 0.66), CD40LG (*r* = 0.59), CD48 (*r* = 0.808), CD70 (*r* = 0.39), CD80 (*r* = 0.625), CD86 (*r* = 0.776), CXCR4 (*r* = 0.473), ICOS (*r* = 0.7), KLRC1 (*r* = 0.448), KLRK1 (*r* = 0.699), LTA (*r* = 0.77), MICB (*r* = 0.659), TMIGD2 (*r* = 0.496), TNFRSF8 (*r* = 0.732), TNFRSF9 (*r* = 0.651), TNFRSF17 (*r* = 0.554), TNFRSF18 (*r* = 0.644), TNFRSF13B (*r* = 0.707), TNFRSF14 (*r* = 0.572) and IL2RA (*r* = 0.439) (**Figure 8A**). The expression of WAS in ccRCC was also significantly associated with immunoinhibitors (*P <* 2.2e-16), such as BTLA (*r* = 0.649), CD244 (*r* = 0.744), CD96 (*r* = 0.777), CSF1R (*r* = 0.72), CTLA4 (*r* = 0.702), IL10 (*r* = 0.516), IL10RB (*r* = 0.419), LAG3 (*r* = 0.761), LGALS9 (*r* = 0.768), PDCD1 (*r* = 0.788), PDCD1LG2 (*r* = 0.53), and TIGIT (*r* = 0.768) (**Figure 8B**). According to these results, WAS may contribute to the regulation of immune interactions and be involved in the regulation of tumor immune escape.

### Correlation between WAS expression and chemokines in ccRCC

**Figure 9:**
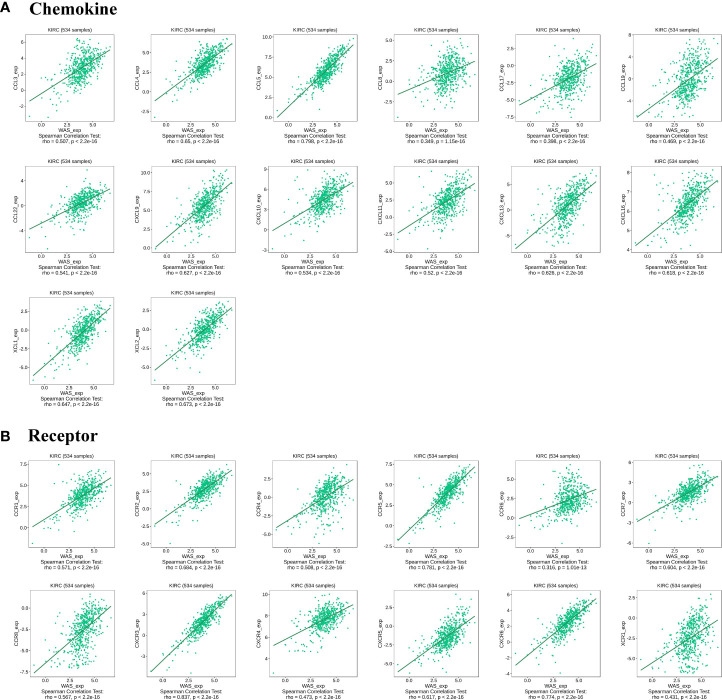
Correlation between WAS and immunoregulatory factors in ccRCC. **(A)** Correlation between WAS expression in ccRCC and chemokines in TISIDB database. **(B)** Correlation between WAS expression and chemokine receptors in ccRCCs in the TISIDB database.

Chemokines are responsible for regulating immune cell infiltration. This study investigated the association between WAS expression and chemokines. The results presented that WAS expression was positively correlated with CCL3 (*r* = 0.507), CCL4 (*r* = 0.65), CCL5 (*r* = 0.798), CCL8 (*r* = 0.349), CCL17 (*r* = 0.398), CCL19 (*r* = 0.469), CCL22 (*r* = 0.541), CXCL9 (*r* = 0.627), CXCL10 (*r* = 0.534), CXCL11 (*r* = 0.52), CXCL13 (*r* = 0.626), CXCL16 (*r* = 0.618), XCL1 (*r* = 0.647*)* and XCL2 (*r* = 0.673) (**Figure 9A**). All *P*-values were < 0.001. Meanwhile, we also demonstrated that WAS expression was considerably positively correlated with chemokine receptors (*P* < 0.001), including CCR1 (*r* = 0.571), CCR2 (*r* = 0.684), CCR4 (*r* = 0.508), CCR5 (*r* = 0.781), CCR6 (*r* = 0.316), CCR7 (*r* = 0.604), CCR8 (*r* = 0.567), CXCR3 (*r* = 0.837), CXCR4 (*r* = 0.473), CXCR5 (*r* = 0.617), CXCR6 (*r* = 0.774) and XCR1 (*r* = 0.431) (**Figure 9B**). These results further demonstrated that WAS may operate as an immunoregulatory factor in ccRCC.

### Immune checkpoint

**Figure 10:**
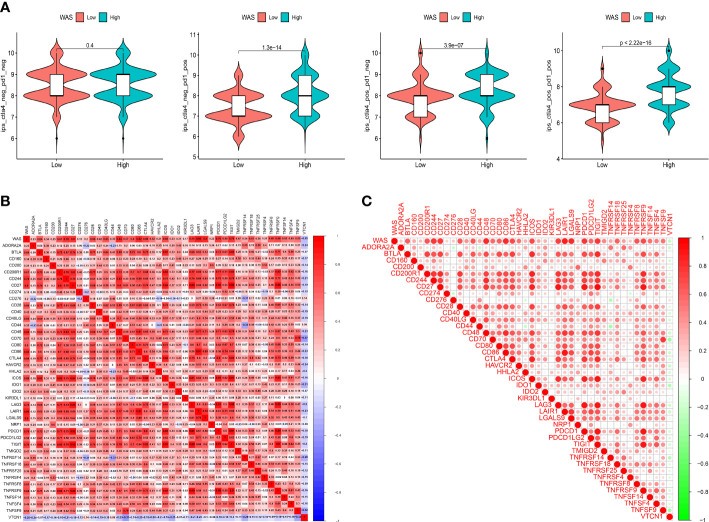
**(A)** Violin plots illustrate the relative probabilities for anti-PD-1 and anti-CTLA-4 treatment responses between high and low WAS expression groups. **(B, C)** The correlation between WAS and 42 immune checkpoints.

This study was further expanded by determining the machine learning-based score (IPS) that predicted patients’ response to ICI treatment. Four subtypes of IPS values (CTLA4_pos_PD1_neg, CTLA4_neg_PD1_pos, CTLA4_neg_PD1_neg and CTLA4_pos_PD1_pos) were carried out to predict the responses to anti-PD1 and anti-CTLA4 treatment among ccRCC patients. We found that the response rate of anti-PD1 and anti-CTLA4 were elevated in high-risk score patients (*P <* 0.001). Similar finding was mirrored for the combination treatment of anti-PD1 and anti-CTLA4 (*P <* 0.001) (**Figure 10A**). A heatmap (**Figures 10B, C**) also showed a positive correlation between WAS and most immune checkpoint genes.

### Drug sensitivity

**Figure 11:**
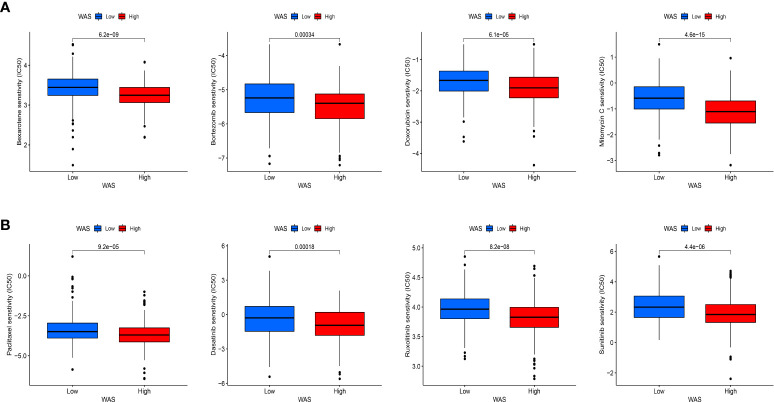
Immunotherapeutic and chemotherapeutic responses for high and low WAS expression patients. **(A)** Boxplots illustrate the immunotherapeutic and chemotherapeutic responses of Bexarotene, Bortezomib, Doxorubicin, and Mitomycin C in different WAS expression patients. **(B)** Boxplots illustrate the immunotherapeutic and chemotherapeutic responses of Paclitaxel, Ruxolitinib, Dasatinib, and Sunitinib in the high and low WAS expression patients.

An IC50 analysis of eight drugs was performed to determine the predictive effect of WAS for treatment responses to chemotherapy and target therapy. The estimated IC50 values of Bexarotene, Bortezomib, Dasatinib, Doxorubicin, Mitomycin C, Paclitaxel, Ruxolitinib, and Sunitinib in high-WAS patients were significantly elevated compared to low-WAS patients, which indicating the high-WAS patients showed a stronger drug resistance (*P* < 0.05) (**Figures 11A, B**). Similarity, patients in low-WAS group were associated with increased sensitivity to other drugs relative to high-WAS patients (*P* < 0.05) (**Figure S1**).

## Discussion

ccRCC is a common urological tumor with an insidious onset, and patients are prone to be diagnosed in middle and advanced stages. In particular, the 5-year survival rate for patients with metastatic ccRCC is less than 10% (35–37). Therefore, it is urge to identify novel biomarkers early for diagnosis and personalized therapies.

In the present study, a bioinformatics investigation was conducted to systematically investigate the clinical significance and expression level of WAS in ccRCC. Our analyses revealed that elevated WAS expression was associated with poor prognosis in ccRCC. Furthermore, our analysis also indicated that WAS expression was significantly associated with the degree of infiltration of multiple immune cells, immunoinhibitors, immunostimulants, receptors and chemokines in ccRCC. Therefore, our study identified WAS as a possible prognostic biomarker associated with immune infiltration in ccRCC. Tumor cells usually obtain energy from glycolysis, during which phosphofructokinase (PFK) is a key enzyme, and TRIM21, an E3-ubiquitinated protein ligase, breaks down PFK (38). Activation of WAS can increase the level of actin expression, promote the chelation of TRIM21 and actin, and reduce the degradation of PFK by TRIM21, thereby enhancing glycolysis and providing more energy for tumor cell metabolism. Several studies have shown that WAS promotes angiogenesis through reorganizing the cytoskeleton and activating MMPs by promoting the migration of vascular endothelial cells (39, 40). The increased expression of WAS, however, can also lead to reduced intercellular adhesion and cell shedding, which is conducive to invasion and metastasis of tumor cells (18). In this study, we assessed WAS expression in ccRCC through online databases including TIMER, GEO, TCGA and UALCAN. We found that WAS mRNA and protein levels were significantly increased in ccRCC compared with paracancerous samples. The HPA database was used to validate protein expression, which found that WAS protein expression was highly elevated in cancer tissues, primarily in renal tumor cells. We validated the abnormal expression of WAS found in the online database by qPCR experiments. Compared with paired paracancerous samples, WAS mRNA levels were elevated in most ccRCC samples. At the same time, WAS gene expression in most ccRCC cell lines was higher than that in the normal renal tubular epithelial cell line. These results suggested that WAS expression level can serve as a potential diagnostic indicator for ccRCC. Furthermore, to confirm whether WAS can be used as a prognostic biomarker, we analyzed the correlation between WAS expression and OS, PFS and DSS in the ccRCC cohort using KM, GEPIA online database and TCGA database. Analysis showed that higher WAS expression was associated with poorer OS and DSS in ccRCC. These results supported our hypothesis that WAS may serve as prognostic biomarker for ccRCC.

Several studies have demonstrated that cancer progression and development are strongly influenced by immune cell infiltration in the TME (41). Cellular immunity is the main mode of tumor immune response. WAS is not only involved in the formation of immune synapses of T lymphocytes, neutrophil migration and phagocytosis of monocytes, but also plays an essential role in the function of Tregs and NK cells (14–16, 42–44). On the other hand, humoral immunity plays a synergistic role in tumor immune response, and WAS affects B lymphocyte function by participating in the process of antigen internalization and presentation (45). Meanwhile, we systematically investigated the relationship between WAS expression and the degree of immune infiltration in ccRCC. Our study showed that the expression of WAS was strongly correlated with TILs such as CD8+ T cells, macrophages, Treg cells, B cells, neutrophils, NK cells, DCs and monocytes. Meanwhile, WAS expression was related to chemokines, receptors, immunostimulatory factors and immunosuppressive factors. In addition, we also analyzed the association between WAS expression and TIL marker genes of ccRCC. Macrophages are a group of differentiated immune cells divided into M1 and M2 macrophages (46). M1 macrophages are induced by Toll-like receptor ligands (bacterial lipopolysaccharides) or Th1 cytokines and exert functions such as bactericidal, pro-inflammatory and antigen presentation, which are associated with favorable prognosis in the cancer context (47–51). M2 macrophages are polarized by Th2 derived cytokines to promote angiogenesis, proliferation (52) and immunosuppression, which is conducive to tumor growth and immune evasion (48). We found that WAS expression correlated with M2 macrophage markers including CD163, VSIG4 and MS4A4A, whereas M1 macrophage markers such as NOS2 and PTGS2 did not. These findings suggest that WAS may potentially be involved in the regulation of immune infiltration in renal cancer.

In addition to this, we also found that elevated WAS expression was closely associated with markers of T cell depletion (PD-1, PD-L1, CTLA-4 and LAG-3) and Tregs markers (FOXP3, CCR8). Up to now, a variety of malignancies, including renal cancer, have been treated with PD1/PDL1 checkpoint blockade therapy, but PD-1 therapy has been found to be less effective in some patients due to PD-1-mediated tumor antigen tolerance poor (53, 54). Hence, it is essential that tumor cells respond better to immune checkpoint inhibitors and cytokines. According to the TISIDB, TIMER and GEPIA databases, we found that increased WAS expression was significantly associated not only with PD-1 and CTLA-4, but also with cellular responses to chemokines. These results suggest that this may be a strategy to enhance the efficacy of immunotherapy by targeting WAS. In conjunction with these results, WAS plays a crucial role in recruiting and regulating TILs in ccRCC, and it is vital to continue investigating the molecular mechanisms and functions of WAS in regulating the tumor microenvironment.

Despite this, our study still has some limitations. First, this study was limited by the fact that partial data were published on online platforms, some clinical information could not be available, such as real-world treatments and responses. Second, bioinformatic analyses did provide insights into the significance of WAS across cancers in terms of cancer immunity, clinical prognosis, and other aspects, but it is still essential to conduct biological validation experiments *in vitro* and *in vivo*.

## Conclusions

In conclusion, this study demonstrated that elevated expression of WAS serves an adverse prognostic factor in ccRCC, and is strongly correlated with aggressive clinical features and unfavorable immune infiltration & immunomodulators. Our findings suggest that WAS could act as a novel as a prognostic predictor of immunotherapy sensitivity. Nevertheless, the mechanism by which WAS mediates the tumorigenesis and progression of ccRCC requires further experiment clarification.

## Data availability statement

The original contributions presented in the study are included in the article/**Supplementary Material**. Further inquiries can be directed to the corresponding author.

## Ethics statement

The studies involving human participants were reviewed and approved by the ethics committee of the affiliated Yantai Yuhuangding hospital of Qingdao university. The patients/participants provided their written informed consent to participate in this study.

## Author contributions

JW and JM constructed this study. GD, TW, SL, and ZZ performed the data analysis, figures plotted, and writing. JW and JM were responsible for the critical reading of the manuscript. All authors contributed to the article and approved the submitted version.

## Supplementary material

Supplementary Figure 1 Boxplots illustrate the immunotherapeutic and chemotherapeutic responses in the high and low WAS expression patients.
