# Supplementary figures and images for: Wiskott-Aldrich syndrome gene as a prognostic biomarker correlated with immune infiltrates in clear cell renal cell carcinoma
Source: Front Immunol. 2023 Apr 12;14:1102824. doi: 10.3389/fimmu.2023.1102824 (PMC10130519; doi:10.3389/fimmu.2023.1102824)

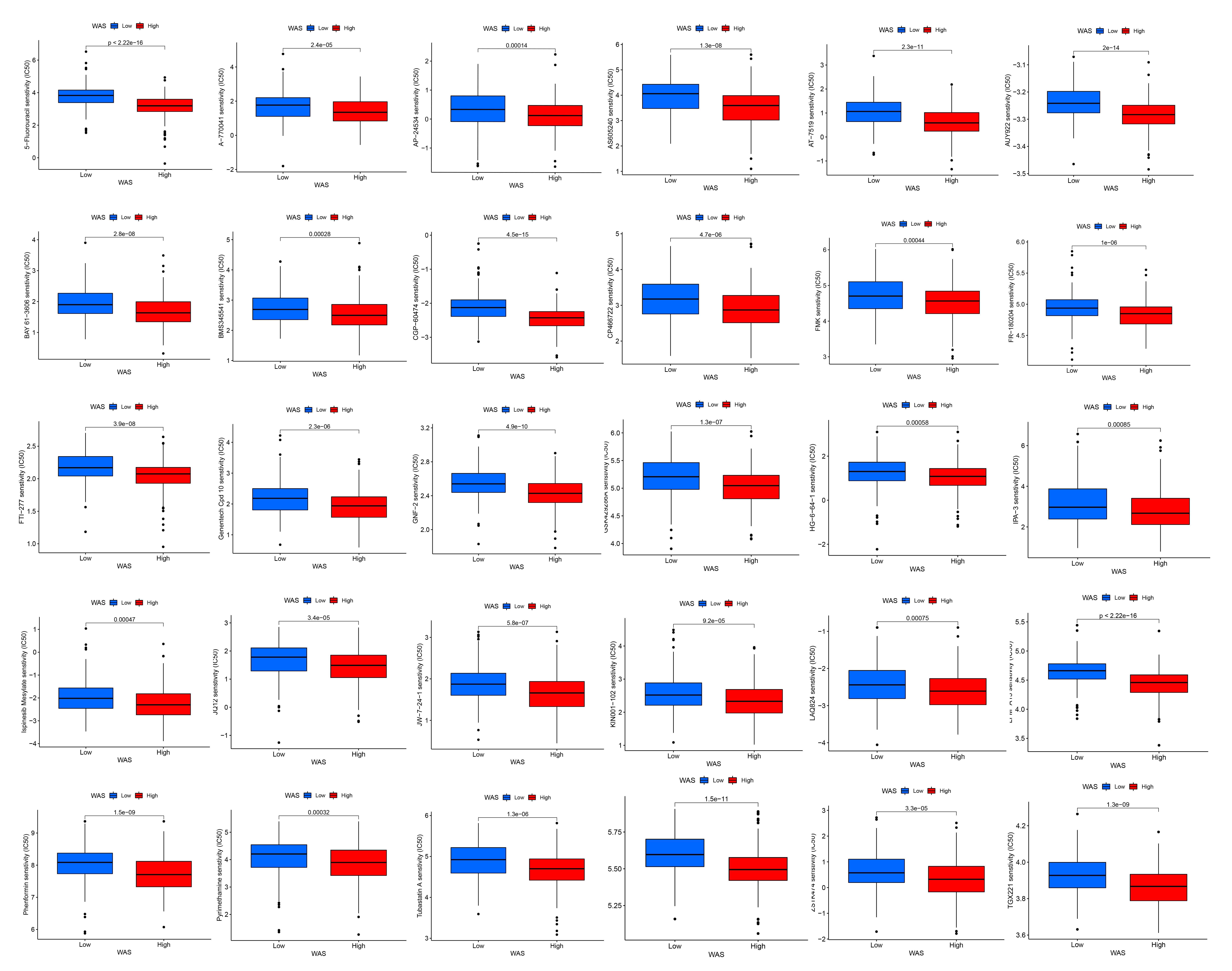

Supplement: Supplementary Figure 1 — Boxplots illustrate the immunotherapeutic and chemotherapeutic responses in the high and low WAS expression patients. [file Image_1.jpg]
